# Lactic acid promotes metastatic niche formation in bone metastasis of colorectal cancer

**DOI:** 10.1186/s12964-020-00667-x

**Published:** 2021-01-21

**Authors:** Jin Qian, Zi-chen Gong, Yi-na Zhang, Hong-hua Wu, Jing Zhao, Li-ting Wang, Li-juan Ye, Da Liu, Wei Wang, Xia Kang, Jun Sheng, Wei Xu, Xi-lin Liu, Juan Wu, Wei Zheng

**Affiliations:** 1 College of Medicine, Southwest Jiaotong University, North Section 1 No.111, Second Ring Road, Chengdu, 610000 People’s Republic of China; 2 Department of Orthopedics, General Hospital of Western Theater Command, Rongdu Avenue No. 270, Chengdu, 610000 People’s Republic of China; 3 Biomedical Analysis Center, Army Medical University, Chongqing, 400038 People’s Republic of China; 4 Department of Pharmacy, General Hospital of Western Theater Command, Rongdu Avenue No. 270, Chengdu, 610000 People’s Republic of China

**Keywords:** Lactic acid, Bone metastasis, Osteoclast precursors, CXCL10, Cadherin-11, PI3K-AKT pathway, Colorectal cancer

## Abstract

**Background:**

To investigate the effect of lactic acid (LA) on the progression of bone metastasis from colorectal cancer (CRC) and its regulatory effects on primary CD115 (+) osteoclast (OC) precursors.

**Methods:**

The BrdU assay, Annexin-V/PI assay, TRAP staining and immunofluorescence were performed to explore the effect of LA on the proliferation, apoptosis and differentiation of OC precursors in vitro and in vivo. Flow cytometry was performed to sort primary osteoclast precursors and CD4(+) T cells and to analyze the change in the expression of target proteins in osteoclast precursors. A recruitment assay was used to test how LA and Cadhein-11 regulate the recruitment of OC precursors. RT-PCR and Western blotting were performed to analyze the changes in the mRNA and protein expression of genes related to the PI3K-AKT pathway and profibrotic genes. Safranin O-fast green staining, H&E staining and TRAP staining were performed to analyze the severity of bone resorption and accumulation of osteoclasts.

**Results:**

LA promoted the expression of CXCL10 and Cadherin-11 in CD115(+) precursors through the PI3K-AKT pathway. We found that CXCL10 and Cadherin-11 were regulated by the activation of CREB and mTOR, respectively. LA-induced overexpression of CXCL10 in CD115(+) precursors indirectly promoted the differentiation of osteoclast precursors through the recruitment of CD4(+) T cells, and the crosstalk between these two cells promoted bone resorption in bone metastasis from CRC. On the other hand, Cadherin-11 mediated the adhesion between osteoclast precursors and upregulated the production of specific collagens, especially Collagen 5, which facilitated fibrotic changes in the tumor microenvironment. Blockade of the PI3K-AKT pathway efficiently prevented the progression of bone metastasis caused by lactate.

**Conclusion:**

LA promoted metastatic niche formation in the tumor microenvironment through the PI3K-AKT pathway. Our study provides new insight into the role of LA in the progression of bone metastasis from CRC.

**Video Abstract**

## Background

Although metabolites derived from cancer cells were once thought to have no effect, it is now well recognized that they contribute to the formation of the metabolic microenvironment and thus regulate intercellular interactions to impact tumor progression and metastasis in broad ways. Lactic acid (LA) is one of the predominant metabolites in the tumor microenvironment. Due to the Warburg effect, a process unique to cancer cells, LA can be produced and accumulate extensively in tumors [1]. It is currently thought that in addition to simply supplying energy, excessive LA performs other physiological functions, such as regulating immunity, metabolism and angiogenesis [2–5].

Previous research has demonstrated that colorectal cancer cells are some of the most active cancer cells that produce LA and that the level of LA is closely associated with the progression of colorectal cancer (CRC). The serum levels of several metabolites, including pyruvic acid, glucose, lactic acid, malic acid, fumaric acid, 3-hydroxybutyric acid, tryptophan, phenylalanine, tyrosine, creatinine and ornithine, were found to be significantly different between CRC patients and control patients and could therefore be good candidates for predicting the prognosis of CRC [6]. One clinical trial further analyzed the relationship between the serum level of LA and metastasis status in CRC, and the results showed that CRC patients with metastasis have higher levels of LA than patients without metastasis [7].

The skeletal system is one of the metastatic sites of CRC, and osteolytic lesions form in affected bone. Although the incidence of bone metastasis from CRC is relatively low compared with that from some other cancers, such as breast cancer and lung cancer, CRC patients with bone metastasis always have a poor prognosis. It have been reported that over half of CRC patients with bone metastasis are in stage 3 or 4 and that the 5-year survival rate of these patients is only 5.7% [8]. Notably, the risk factors for a low survival rate in CRC patients are cancer cells in the bone marrow, initial bone metastasis and bone metastasis from colon cancer [8–10].

Bone is thought to be unsuitable for the survival of cancer cells due to the high content of inorganic minerals. Bone metastasis of cancer cells requires assistance from a variety of other cell types to facilitate the colonization of cancer cells. It is widely accepted that abnormal activation of osteoclasts is critical for the formation of osteolytic lesions in bone metastases from a variety of cancers, including CRC, to create a metastatic niche [11–14]. Unfortunately the detailed metabolite-mediate communication that occurs between CRC cells and osteoclasts or their precursors is still unclear. Considering the abundant accumulation of lactic acid that occurs in CRC, it is important to explore whether and how LA regulates osteoclasts and contributes to the progression of CRC bone metastasis.

In our study, we demonstrated that LA facilitates metastatic niche formation in bone metastasis from CRC through overexpression of CXCL10 and Cadherin-11 in primary osteoclast precursors. First, LA activates the PI3K-AKT-CREB pathway in CD115(+) precursors to upregulate the expression of CXCL10. Then, CXCL10 stimulates the recruitment of CD4(+) T cells into metastatic sites and induces the production of RANKL, which ultimately promotes the osteoclastogenesis of CD115(+) precursors. Second, LA can upregulate the expression of Cadherin-11 in CD115(+) precursors through activation of mTOR. The overexpression of Cadherin-11 inhibits the recruitment of CD115(+) precursors but enhances adhesion between them. Moreover, collagen deposition can be selectively enhanced by overexpression of Cadherin-11. Our results provide new insight into the regulation of osteoclast accumulation in the tumor microenvironment by LA and shows the critical roles of lactic acid in metastatic niche formation and the poor prognosis of bone metastasis from CRC.

## Materials and methods

### Animal experiments

All animal experiments and procedures were approved by the Institutional Animal Care and Use Committee at the General Hospital of Western Theater Command. Male C57BL/6 mice aged 6 to 8 weeks were used for experiments. To establish the bone metastasis model, 500,000 MC-38 cells were injected into the tibia following a previously described standard method [15]. Lactate (2.5 mg/kg) was injected intraperitoneally for 3 continuous days. For some experiments, the PI3K inhibitor BYL719 (25 mg/kg/d) was administered by oral gavage 5 days per week. AMG-487 (5 μg/g) was injected twice a day. CXCL10 neutralizing antibodies (50 μg, Peprotech) were intraperitoneally injected three times weekly.

### Cell isolation and FACS

Because CD115(+) RANK(−) cells (referred to as CD115(+) precursors hereafter) in the bone marrow are considered the primary osteoclast precursors [12], we performed in vitro experiments with sorted CD115(+) RANK(−) osteoclast precursors. In some experiments, primary CD4(+) T cells in the bone marrow were sorted as well. Briefly, FACS was performed as follows: the bone marrow was flushed with sterile PBS. The suspension was filtered with 100 μm filter strainers and centrifuged at 500 g/min. The bone marrow cells were cultured with primary antibodies for 30 min on ice in the dark. After washing with wash buffer 2 times, the stained cells were sorted by a FACSAria III flow cytometer (BD Biosciences). The antibodies used for FACS were anti-mouse CD115-APC (Biolegend) and anti-mouse RANK-PE (Biolegend) for osteoclast precursors, anti-mouse CD45-FITC (Biolegend), anti-mouse CD3, anti-mouse CD90.2-PE (Biolegend), anti-mouse CD45R-APC (Biolegend) and anti-mouse CD4-APC-cy7 (Biolegend) antibodies for CD4(+) T cells.

### Cell culture

Sorted primary CD115(+) precursors were cultured in α-minimal essential medium (MEM) containing 10% FBS, 1% penicillin-streptomycin solution and 50 ng ml^− 1^ colony stimulating factor (M-CSF, Abcam). After 3 days, the medium was replaced with fresh medium. LA was added at a concentration of 100 μg/ml to stimulate the precursors. To inhibit the expression of Cadherin-11, the primary osteoclast precursors were transfected with Cadherin-11 siRNA using INVI DNA RNA Transfection reagent (Invigentech) for 24 h. The sequence of Cadherin-11 siRNA was CCAATGGACCAAGATTTAT. In some experiments, primary cells were treated with BYL719 (2.5 μM), GSK690693 (25 nM), 666–15 (50 nM), rapamycin (20 μM), or AMG-487 (30 μM).

### Cell migration assay

After being transfected with siRNA or scRNA, the cells were seeded in the upper chamber. Lactic acid (100 μg/ml) was added to the culture medium in the lower chamber. The cells were stained with crystal violet after culturing for 12 h.

### RT-PCR analysis

Total RNA was isolated and analyzed using TRIzol reagent. Then, the RNA was reverse transcribed into cDNA by using a RevertAid First Strand cDNA Synthesis kit (Thermo Fisher Scientific) following the manufacturer’s procedures. The mRNA levels were normalized to the level of GAPDH. Relative target gene expression was calculated using the 2-ΔΔCq method. The primer sequences used for PCR were as follows: *Gapdh* (RE: TGTAGACCATGTAGTTGAGGTCA; FW: AGGTCGGTGTGAACGGATTTG), *Cxcl10* (RE:CCACGTGTTGAGATCATTGCC; FW: TCACTCCAGTTAAGGAGCCC), *Rankl* (RE:TACTTTCGAGCGCAGATGGAT; FW:CTGCAGGAGTCAGGTAGTGTG), *Cdh11* (RE: CCAATCAGATGGGTGGAGCA*; FW:*CTCCGCAGTCAGCTTCTTCT*), Col5a2 (RE: GTACCACTGGGCAAAGAGGA; FW:*CTTTTCCTGGTGTACCCGCT*), Col3a1 (RE:* TCCTGGTGGTCCTGGTACTG*; FW:*AGGAGAACCACTGTTGCCTG*), Col6a1 (RE:* CCTGGGGATCTTGGACCAGT*; FW:*TTGCCTTTCTCGCCCTTGTA*), Col6a3 (RE:* GCCCAACAGCATGGAGATGT*; FW:*CTTCCCAGCACTCCAAGAGG*), and* CXCR3 *(RE:* GCCATGTACCTTGAGGTTAGTGA*; FW:*ATCGTAGGGAGAGGTGCTGT*).*

### Western blotting

Proteins (20 μg) were separated on SDS-PAGE gels. Then, the proteins were transferred to polyvinylidene difluoride (PVDF) membranes (Bio-Rad Laboratories). The PVDF membranes were then blocked with 5% BSA diluted in TBS for 1 h at room temperature. Primary antibodies against CXCL10 (R&D), RANKL (Abcam), Cadherin-11 (Invitrogen), mTOR (Abcam), phosphorylated mTOR (Abcam), CREB (Abcam), phosphorylated CREB (Abcam), AKT (Abcam), phosphorylated AKT (Abcam), PI3K (Abcam), phosphorylated PI3K (Abcam) and GAPDH (Abcam) were then added according to the manufacturers’ protocols. The samples were agitated at 4 °C overnight. HRP-conjugated rabbit anti-mouse IgG (Abcam), mouse anti-rabbit IgG (Abcam) and goat anti-mouse IgG (Abcam) secondary antibodies were added and incubated at room temperature for 2 h. Densitometric analysis was performed using the ChemiDoc Touch Imaging System (Bio-Rad Laboratories).

### Histochemistry, immunofluorescence and imaging

The hindlimbs were removed from the mice at the time of sacrifice, and the bones were fixed in 4% paraformaldehyde (PFA) for 4 days. Then, the samples were washed and decalcified in 10% EDTA for 2 weeks and embedded in paraffin. For histochemistry, decalcified tibial sections were stained with tartrate-resistant acid phosphatase (TRAP) (Wako), safranin O-fast green or H&E following the manufacturer’s protocols. For immunohistochemistry, the sections were subjected to antigen retrieval with 0.1% trypsin (Invitrogen), washed and blocked at 37 °C for 1 h. Then, the samples were incubated with primary antibodies overnight followed by secondary antibodies. For cytochemistry, primary CD115(+) precursors were seeded in 24-well plates and stimulated as appropriate. Then, the cells were fixed in 4% paraformaldehyde (PFA) for 15 min, washed and blocked at 37 °C for 30 min. Then, the cells were incubated with primary antibodies overnight followed by secondary antibodies. A CD115 antibody diluted 1:100 (Novus Biologicals), Ki67 antibody diluted 1:100 (Abcam), TRITC-conjugated phalloidin diluted 1:200 (Yeason), cadherin-11 antibody diluted 1:50 (Invitrogen), and collagen 5 antibody diluted 1:100 (Abcam) were used. The secondary antibodies used were Alexa Fluor 488-conjugated donkey anti-rabbit, Alexa Fluor 555-conjugated donkey anti-rat, and Alexa Fluor 488-conjugated donkey anti-mouse secondary antibodies (Jackson ImmunoResearch). The samples were counterstained with Hoechst 33342. Confocal images of bone sections were captured using the TCS SP8 confocal laser scanning microscope system (Leica Microsystems).

### In vitro osteoclastogenesis assays

FACS-sorted primary CD115(+) precursors were seeded in 96-well plates or 24-well plates. The cells were cultured in α-minimal essential medium (MEM) containing 10% FBS and 1% penicillin-streptomycin solution with CSF for 2 days. To induce osteoclast differentiation, the cells were exposed to induction medium consisting of MEM with 10% FBS, RANKL (50 ng/ml) and CSF (50 ng/ml) for at least 4 days. For tartrate-resistant acid phosphatase (TRAP) staining, induced cells were fixed in 4% paraformaldehyde for 10 min and then stained with TRAP staining solution according to the manufacturer’s instructions (Wako). Relative TRAP activity was measured by colorimetric analysis according to the manufacturer’s instructions (Keygen). Images were captured by an IX81 fluorescence microscope (Olympus).

### Flow cytometry analysis

Bone marrow cells were flushed from the tibia and processed as described above, and then the cells were incubated in the appropriate antibodies for 30 min at 4 °C. The antibodies used for flow cytometry analysis were anti-mouse CD45-FITC (Biolegend), anti-mouse CD3, anti-mouse CD90.2-PE (Biolegend), anti-mouse CD45R-APC (Biolegend), anti-mouse CD4-APC-cy7 (Biolegend), anti-mouse CD115-APC (Biolegend), anti-mouse RANK-PE (Biolegend), and anti-mouse Cadherin-11 (Invitrogen) antibodies. The BrdU assays was performed using the Phase-Flow™ FITC BrdU Kit (Biolegend). Briefly, cells were treated with BrdU (0.5 μL/mL) for 3 h. Then, the cells were collected, fixed and permeabilized. After treatment with DNase for 1 h at 37 °C, the samples were incubated with a BrdU antibody conjugated to Alexa Fluor 488 for 30 min. For in vitro apoptosis analysis, the samples were resuspended in 400 μL of staining buffer and stained with Annexin V (5 μL) for 15 min followed by PI (10 μL) for 5 min (Solarbio). The stained samples were analyzed by flow cytometry (BD FACSCalibur, BD Biosciences). The data were analyzed by using FlowJo v10 software (FlowJo, LLC).

### Indirect coculture assay

The CD4(+) T cells were sorted and cultured in the presence of CXCL10 with/without AMG-487 for over 3 days, after which the culture medium was replaced with DMEM without FBS for 24 h. Then, the conditioned medium was collected and stored in a deep freezer. For the indirect coculture assay, the collected conditioned medium was added to freshly prepared medium at a 1:1 ratio and used to stimulate CD115 (+) precursors, which were used in subsequent experiments.

### Lactic acid assay

The lactate concentration detection kit (Solarbio) was used to test the lactate concentration in conditioned medium and bone marrow following the manufacturer’s protocol. Briefly, for conditioned medium, 100 μL medium was added to the extraction solution, and the mixture was centrifuged at 12,000 rpm for 10 min. Then, 0.8 mL supernatant was added to 0.15 mL extraction solution 2 and centrifuged at 12,000 rpm for another 10 min. Then, 50 μL supernatant was mixed with solution 1, solution 2, solution 5 and incubated for 20 min at 37 °C. Then, the mixture was mixed with solution 6 and allowed to undergo coloration for 20 min at 37 °C. After centrifugation, the precipitate was added to 1 mL ethanol and detected at 570 nm. For bone marrow, 1 mL extraction solution 1 was injected into the medullary space of one leg 2–3 times, and the subsequent procedures were the same as the methods for conditioned medium.

### RNA-seq analysis

Osteoclast precursors were treated with or without lactic acid in the presence of CSF (50 ng/ml) for 48 h. Total RNA was isolated using TRIzol reagent (Invitrogen). A TruSeq™ RNA sample preparation kit (Illumina) was used to prepare the RNA-seq transcriptome library and sequenced with Illumina HiSeq xten (2 × 150 bp read length). The profiling data were analyzed on the free online platform Majorbio Cloud Platform (www.majorbio.com).

### Statistical analysis

The results are shown as the means ± SE or SD as required. Student’s *t*-test was used to compare two groups. For more than two groups, one-way analysis of variance (ANOVA) was used. Statistical significance was considered at *P* < 0.05. For some data, values not sharing a common small letter differ significantly (*p* < 0.05); *p < 0.05, **p < 0.01, ***p < 0.001. All experiments were repeated at least 3 times.

## Results

### Lactic acid facilitates the progression of osteolytic lesions in bone metastasis from CRC

**Fig. 1:**
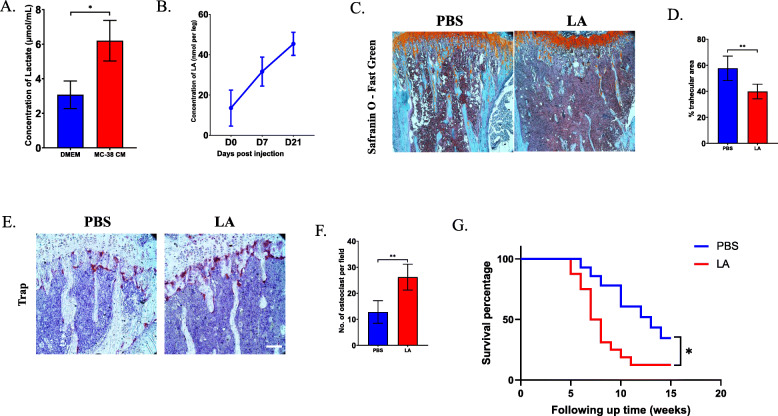
LA facilitates the progression of bone metastasis from CRC. **a** The concentration of LA in MC-38 conditional media was tested by ELISA assay after cultured for 3 days. **b** The concentration of LA in bone marrow at specific timepoints after injection of MC-38 cells was tested by ELISA assay. **c** Safranin O and fast green staining was performed to detect the trabecular area between PBS treated group and LA treated group at 2 weeks post injection of MC-38 (Scale bar = 50 μm). **d** Quantification of trabecular area in (**c**). **e** TRAP staining showed the osteoclast accumulation in trabecular area in PBS-treated group and LA-treated group at 2 weeks after injection of MC-38 (Scale bar = 50 μm). **f** Quantification of osteoclast numbers in (**e**). **g** Survival analysis in LA-treated group vs. control group in bone metastasis model of CRC. *p < 0.05, **p < 0.01, ***p < 0.001

To confirm that LA can be produced by MC-38 cells, a murine colorectal cancer cell line, cells were cultured for 3 days, and the concentration of LA in the culture medium was examined by ELISA. As expected, the level of LA was elevated more than 2-fold compared with that in the control group (Fig. 1a). Then, we measured the change in the LA level in the metastatic model in vivo. After intratibial injection of MC-38 cells, the concentration of LA increased gradually over time (Fig. 1b).

Next, we investigated the effect of LA on the progression of bone metastasis from CRC in vivo. Interestingly, the severity of osteolytic lesions was significantly increased after administration of LA (Fig. 1c), and the percentage of trabecular area was decreased nearly 20% at 2 weeks (Fig. 1d). Consistently, the number of osteoclasts was increased markedly after administration of LA (Fig. 1e and f). Considering that the severity of osteolytic lesions correlated with the survival rate of bone metastasis, we analyzed the overall survival of the group treated with LA compared to that of the group treated with PBS. As expected, the LA-treated group showed poorer prognosis than the PBS-treated group (Fig. 1g).

### Lactic acid facilitates the accumulation of osteoclasts through overexpression of CXCL10 in CD115(+) precursors

To determine the cause of the accumulation of osteoclasts, we analyzed the effect of LA on the proliferation, apoptosis and differentiation of OC precursors. Since CD115(+) precursors were considered osteoclast precursors in vivo, we first sorted the CD115(+) precursors via FACS and cultured them in vitro (Figure S1A). After 3 days of treatment with LA, the percentage of Ki67-positive OC precursors was not significantly changed compared with that in the control group (Figure S1B). The results of the BrdU assay suggested that the proliferation of OC precursors was not affected by different concentrations of LA with/without CSF (Figure S1C and S1D). Then, we explored whether LA impacted the apoptosis of OC precursors. The Annexin-V/PI assay showed that the percentage of OC precursors in vitro was not changed by LA treatment for 3 days (Figure S1E). Next, the effect of LA on the differentiation of OC precursors was investigated. After CD115(+) precursors were treated with LA plus RANKL for 4 days, there was no significant difference in TRAP activity between the LA-treated group and the control group (Figure S1F). Together, these data showed that LA did not directly affect the proliferation, apoptosis or differentiation of OC precursors.

**Fig. 2:**
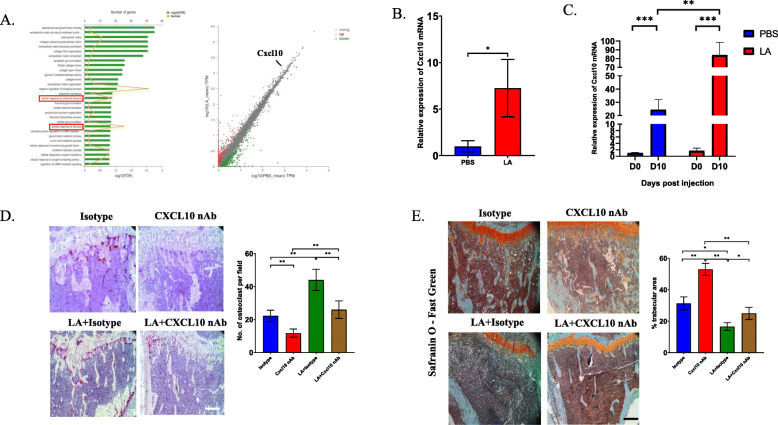
LA induces the expression of CXCL10 in osteoclast precursors to promote the formation of osteolytic formation. **a** Cellular pathways enriched in RNA-Seq analysis and scatterplot illustration showed differently expressed genes in CD115(+) precursors in LA-treated group comparing with that genes expressing in control group. **b** RT-PCR analysis showed the mRNA level of Cxcl10 in CD115(+) precursors treated by LA for 72 h comparing with treated by PBS. **c** RT-PCR analysis detected the mRNA expression of Cxcl10 in freshly sorted CD115(+) precursors from bone marrow after injection of MC-38 cells at 0 day and 10 day with/without treatment with LA. **d** Trap staining revealed number of Trap(+) cells at 14 day after injection of CRC cells and administration of LA with/without CXCL10 neutralizing antibody, and the quantification of the number of osteoclasts (Scale bar = 50 μm). **e** Safranin O and fast green staining showed the trabecular area at 14 day after injection of MC-38 cells and treated by LA with/without CXCL10 neutralizing antibody, and quantification of percentage of trabecula area (Scale bar = 50 μm). *p < 0.05, **p < 0.01, ***p < 0.001

Then, we investigated whether LA indirectly impacts the accumulation of OCs. RNA-seq analysis showed changes in many pathways in the LA-treated group compared with the control group. Among the altered pathways, we noticed two pathways, “cellular response to chemical stimulus” and “cellular response to stimulus”, that were significantly changed. Interestingly, the two pathways contained a common chemokine, namely, CXCL10, which was significantly upregulated in the LA-treated group compared to the control group (Fig. 2a). Other cytokines/chemokines that were significantly changed after treatment with LA included CXCL9, CXCL5, and IL27 and can be found in Supplemental Table 1 (Table S1). RT-PCR analysis confirmed that the mRNA level of CXCL10 in CD115(+) precursors was increased more than 5-fold after treatment with LA in vitro (Fig. 2b). Moreover, we also found that the expression of CXCL10 was upregulated over time in CD115(+) precursors after intratibial injection of MC-38 and that LA significantly enhanced the expression of CXCL10 in vivo (Fig. 2c). Next, we explored whether CXCL10 contributes to the accumulation of OCs in bone metastasis from CRC. In the bone metastasis model of CRC, TRAP staining showed that the number of osteoclasts significantly decreased after blockade of CXCL10 and that administration of LA markedly increased the number of osteoclasts (Fig. 2d). Consistently, safranin O and fast green staining showed that LA caused the most severe bone resorption in bone metastasis from CRC and that the trabecular area was preserved after blockade of CXCL10 (Fig. 2e). These results implied that LA may promote osteolytic lesion formation through upregulating the expression of CXCL10.

### CXCL10 recruits CD4(+) T cells to promote the osteoclastogenesis of CD115(+) precursors in bone metastasis from CRC

We next induced the differentiation of CD115(+) precursors in vitro by treatment with CXCL10. Unexpectedly, TRAP staining and TRAP activity analysis showed that both the number of TRAP(+) cells and TRAP activity were not obviously different in the CXCL10-treated group compared with the control group, indicating that CXCL10 did not directly contribute to the differentiation of OC precursors (Figure S2A and S2B). Moreover, RT-PCR analysis revealed that the mRNA level of CXCR3, the receptor of CXCL10, was not significantly changed by CXCL10 treatment, suggesting that CXCL10 did not directly act on CD115(+) precursors (Figure S2C).

**Fig. 3:**
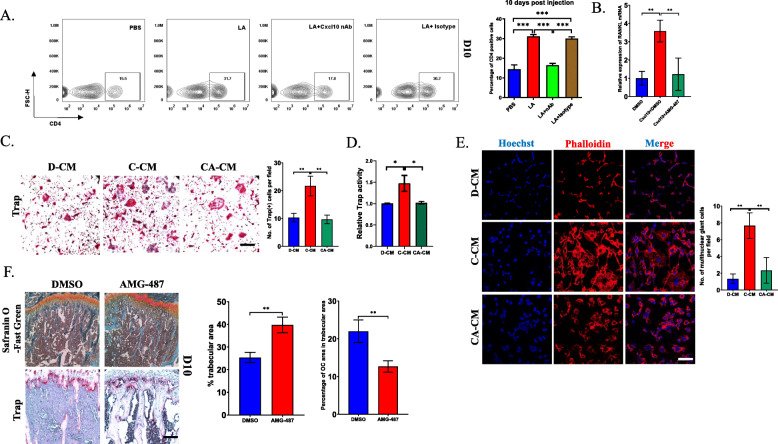
CXCL10 recruits CD4+ T cells to promote the osteoclastogenesis of CD115(+) precursors. **a** Flow cytometry analysis detected the percentage of CD4(+) T cells in bone marrow at 10 day after injection of MC-38 cells into tibia and treatment with LA as well as CXCL10 neutralizing antibody. **b** CD4(+) T cells were sorted from bone marrow and then was stimulated by CXCL10 recombinant protein with/without AMG-487 for 72 h, the mRNA level of RANKL was detected by RT-PCR analysis. **c** After CD4(+) T cells were treated by CXCL10 with/without AMG-487, conditional medium was collected. Then CD115(+) precursors were treated by conditional medium for 6 days in the presence of RANKL (50 ng/mL) and M-CSF (50 ng/mL). TRAP staining was used to analyze the osteoclastogenesis of CD115(+) precursors, and the number of osteoclasts in each group was quantified (Scale bar = 50 μm). D-CM: conditional medium after treated by DMSO; C-CM: conditional medium after treated by CXCL10; CA-CM: conditional medium after treated by CXCL10 and AMG-487. **d** TRAP activity analysis tested the Trap activity in each group of (C). **e** Immunofluorescence showed the multinuclear giant cells after treated by each conditional medium for at least 4 days in the presence of RANKL and M-CSF, and the quantification of the number of multinuclear giant cells (Scale bar = 50 μm). *p < 0.05, **p < 0.01, ***p < 0.001

As an important chemokine, CXCL10 can enhance the recruitment of CD4(+) T cells, as reported in previous studies [16–20]. Thus, we thus examined the percentage of CD4(+) T cells after treatment with a CXCL10 nAb in bone metastasis from CRC. Interestingly, flow cytometry analysis showed that the percentage of CD4(+) T cells was increased approximately 2.5-fold in the LA-treated group compared with the control group at 10 days and decreased significantly after treatment with the CXCL10 nAb (Fig. 3a). Since it has been reported that CD4(+) T cells can produce RANKL to induce the differentiation of osteoclast precursors [20], we sorted CD4(+) T cells in the presence of CXCL10 recombinant protein with/without AMG-487, an antagonist of CXCR3, for 3 days. RT-PCR showed that CXCL10 enhanced the expression of RANKL in CD4(+) T cells, while AMG-487 can reverse the elevation of RANKL expression stimulated by CXCL10, indicating that CXCL10 can promote the overexpression of RANKL in CD4(+) cells via CXCR3 (Fig. 3b). To further confirm the effect of CD4(+) T cells on the osteoclastogenesis of CD115(+) precursors, we collected conditioned medium from CD4(+) cells treated with CXCL10 with/without AMG-487 to stimulate CD115(+) precursors. After treatment for 6 days, TRAP staining revealed that the number of TRAP(+) giant cells was markedly increased in the CXCL10-treated conditioned medium (C-CM) group compared with the control (D-CM) group and that the number of osteoclasts was obviously decreased obviously in the CXCL10 plus AMG-487-treated conditional medium (CA-CM) group compared to the control group. The results of TRAP activity analysis were consistent with these findings (Fig. 3c and d). Furthermore, immunofluorescence analysis revealed that there were many more multinuclear giant cells in the C-CM group than in the control (D-CM) group and the CXCL10 plus AMG-487-treated (CA-CM) group (Fig. 3e). Since we found that CXCL10 promoted the osteoclastogenesis of CD115(+) precursors indirectly via CXCR3, we injected AMG-487 to explore whether the osteolytic lesion is attenuated after inhibition of CXCR3. As expected, the trabecular area was preserved in the AMG-487-treated group, while the number of OCs was obviously decreased compared to that in the control group (Fig. 3f).

### LA elevates the expression of CXCL10 through activation of the PI3K-AKT-CREB pathway in CD115(+) precursors

**Fig. 4:**
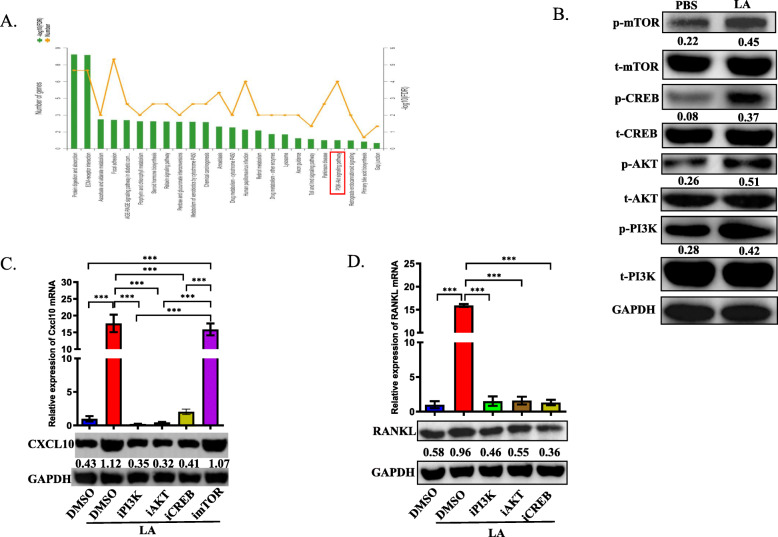
The activation of PI3K-AKT-CREB pathway at least partly regulates the expression of CXCL10 in osteoclast precursors. **a** GO ontology analysis showed the enriched pathways in transcriptomic profiling in LA-treated group comparing with control group. **b** Western blotting analysis showed the activation of PI3K-AKT pathways, including the total protein levels and phosphorylated levels of mTOR, CREB, AKT and PI3K. **c** RT-PCR analysis and Western blotting showed the mRNA expression and protein expression of CXCL10 in CD115(+) precursors treated by LA with/without each antagonist of PI3K-AKT pathway for 72 h in vitro. **e** Conditional medium was collected from CD115(+) precursors after stimulated by LA with/without each antagonist of PI3K-AKT pathway, then CD4(+) T cells were treated by conditional medium for 48 h. RT-PCR analysis and Western blot detected the mRNA expression and protein level of RANKL in each group. *p < 0.05, **p < 0.01, ***p < 0.001

To further investigate the regulatory effect of LA on the expression of CXCL10 in CD115(+) precursors, we analyzed the transcriptomic profile of LA-treated CD115(+) precursors compared with that of the control group. GO ontology analysis showed that the PI3K-AKT pathway was changed in the LA-treated group (Fig. 4a). Since the PI3K pathway has been reported to regulate the expression of CXCL family members [21, 22], we first examined whether LA can activate the PI3K pathway. Interestingly, the protein levels of phosphorylated PI3K, AKT and two main transcriptional factors, namely, CREB and mTOR, in CD115(+) precursors were upregulated significantly in the LA-treated group compared to the control group, indicating that LA can activate the PI3K-AKT pathway in osteoclast precursors (Fig. 4b). A previous study indicated that one of the transcription factors of the PI3K-AKT pathway, CREB, may be involved in the transcription of CXCL10 [23]. To verify this, we treated CD115(+) precursors with BYL719, GSK690693, 666–15 and rapamycin, which are a PI3K antagonist, AKT antagonist, CREB antagonist and mTOR antagonist, respectively, to test their effects on the expression of CXCL10. RT-PCR analysis and Western blot analysis showed that the mRNA level and protein level of CXCL10 was downregulated by PI3K, AKT and CREB inhibitors but not an mTOR inhibitor, indicating that the expression of CXCL10 was regulated by the PI3K-AKT-CREB pathway (Fig. 4c). Then, conditioned medium from cells treated with these inhibitors was collected to stimulate CD4(+) T cells. As expected, the mRNA level and protein level of RANKL in CD4(+) T cells was significantly decreased in cells cultured with inhibitor-treated CM compared to cells cultured in LA-treated CM (Fig. 4d). These data demonstrated that the activation of the PI3K pathway caused by LA upregulates the expression of CXCL10 in CD115(+) cells; moreover, inhibiting the activation of components of the PI3K pathway in CD115(+) precursors can efficiently decrease the expression of RANKL in CD4(+) T cells.

### Lactic acid facilitates the colonization of OC precursors and collagen deposition through upregulating the expression of Cadherin-11

**Fig. 5:**
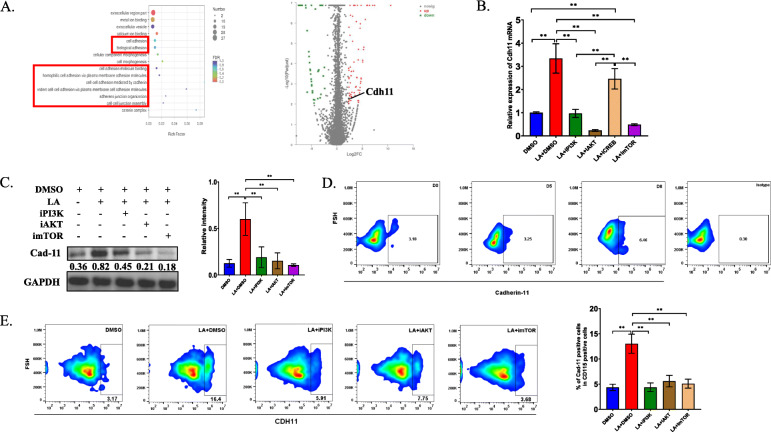
LA induces the overexpression of Cadherin-11 in osteoclast precursors through PI3K-AKT-mTOR pathway. **a** KEGG pathway analysis of differentially regulated targets (left) and the expression of Cdh11 (right). **b** RT-PCR analysis showed the mRNA expression of Cadherin-11 in CD115(+) precursors treated by LA with/without each antagonist of PI3K-AKT pathway in vitro for 72 h. **c** Western blots analysis showed the protein level of Cadherin-11 in CD115(+) precursors after treated by LA as well as each antagonist of PI3K-AKT-mTOR pathway, respectively, and the quantification of relative intensity. **d** Flow cytometry analysis showed the percentage of Cadherin-11(+) cells in CD115(+) precursors at specified timepoints after injection of MC-38 cells. **e** After intratibially injecting MC-38 cells, LA with/without each antagonist of PI3K-AKT-mTOR pathway was injected. After 5 days, the percentage of Cadherin-11(+) cells in CD115(+) precursors was analyzed by flow cytometry, and the quantification of the percentage of Cadherin-11(+) precursors. *p < 0.05, **p < 0.01, ***p < 0.001

RNA-seq profiling also showed that several cell adhesion pathways were changed after treatment with LA (Fig. 5a). We screened adhesion-related proteins and found that Cdh11, which encodes Cadherin-11, was significantly changed in the LA-treated group compared to the control group (Fig. 5a). Previous studies have suggested that the expression of Cadhein-11 is associated with the PI3K pathway, but the causal relationship is unclear [24–27]. To confirm this finding, RT-PCR and Western blotting were performed to analyze the expression of Cadherin-11 after treatment with LA. As expected, both the mRNA level and protein level of Cadherin-11 in primary OC precursors were upregulated after treatment with LA, and Cadherin-11 was inhibited by PI3K, AKT and mTOR antagonists but not by a CREB antagonist (Fig. 5b and c), indicating that the overexpression of Cadherin-11 was regulated by the PI3K-AKT-mTOR pathway.

Next, we further explored the expression of Cadhein-11 in CD115(+) precursors over time after intratibial injection of MC-38. Flow cytometry analysis showed that the percentage of Cadherin-11(+) cells remained stable before 5 days and was increased approximately 2-fold after 8 days (Fig. 5d). Then, we investigated whether the expression of Cadherin-11 in CD115(+) precursors is impacted by LA and the PI3K pathway. Interestingly, the percentage of CD115(+) precursors that were Cadherin-11(+) significantly increased in the group treated with LA 5 days after intratibial injection of MC-38 compared with the control group. However, the percentage of these cells was decreased after injection of PI3K pathway inhibitors (Fig. 5e). Our results showed that LA can promote the overexpression of Cadherin-11 in CD115(+) precursors both in vivo and in vitro and that this effect is regulated by activation of the PI3K-AKT-mTOR pathway.

### Cadherin-11 regulates LA-mediated adhesion and selective collagen production in CD115(+) precursors

**Fig. 6:**
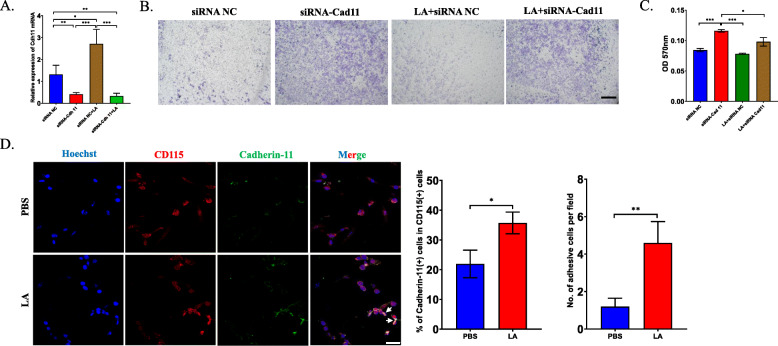
The overexpression of Cadherin-11 enhances the adhesion ability of osteoclast precursors. **a** RT-PCR analysis tested the mRNA level of Cadherin-11 in CD115(+) precursors to verify the efficiency of Cadherin-11 siRNA. **b** Transwell assay showed the mobility of CD115(+) precursors after transfected with Cadherin-11 siRNA or negative control in the presence of LA or not for 12 h. **c** The quantification of Transwell assay in (**b**) by using absorbency analysis. **d** Immunofluorescence analysis showed Cadherin-11 expression in CD115(+) precursors after treated by LA for 72 h (left), the percentage of Cadherin-11 and CD115 dual-positive cells in total cells was calculated (middle) and the quantification of adhesive CD115(+) precursors (right). *p < 0.05, **p < 0.01, ***p < 0.001

It is well recognized that Cadherin family members participate in cell recruitment and adhesion, and we explored whether Cadherin-11 contributes to the accumulation of osteoclasts mediated via LA. First, we synthesized siRNA against cadherin-11 (siRNA-Cdh11). RT-PCR revealed that siRNA-Cdh11 efficiently downregulated the mRNA level of Cadherin-11. In addition, LA upregulated the expression of Cadherin-11, which is consistent with our previous findings (Fig. 6a). Then, we explored whether recruitment of CD115(+) precursors is affected by downregulation of Cadhein-11. Interestingly, recruitment analysis revealed that the mobility of CD115(+) precursors was markedly increased after treatment with Cadherin-11 siRNA, and that this change was reversed in the presence of LA (Fig. 6b). Absorbance measurements at 570 nm supported the results of the recruitment analysis (Fig. 6c). The above data indicated that Cadherin-11 negatively regulates the recruitment of CD115(+) precursors and that the impairment in Cadherin-11 recruitment can be rescued by LA.

Previous studies found that two cells expressing Cadherin-11 can anchor each other [28]; thus, we further investigated whether Cadherin-11 mediates the adhesion of CD115(+) cells. As expected, immunofluorescence showed that the expression of Cadherin-11 was upregulated in CD115(+) precursors in the LA-treated group compared to the control group; moreover, Cadherin-11 was expressed at a higher level at the junction between two adhesive cells than at the junction between nonadhesive cells (Fig. 6d). Our findings suggest that Cadherin-11 may mediate the adhesion of CD115(+) precursors with each other.

**Fig. 7:**
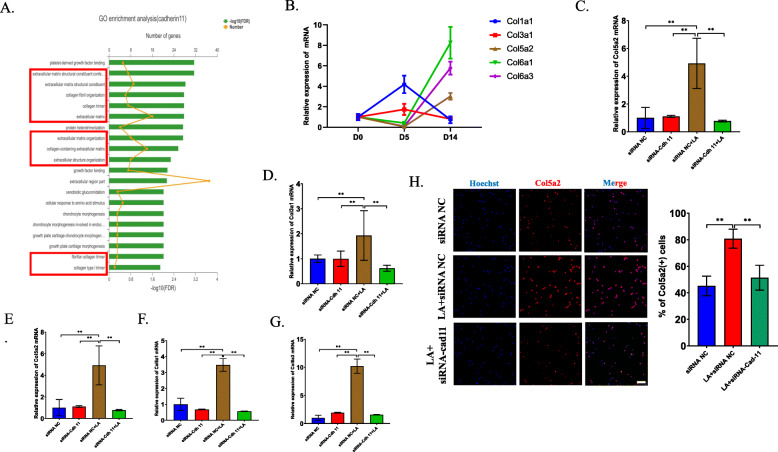
LA facilitates the fibrosis through upregulating the expression of Cadherin-11. **a** Go enrichment analysis showed pathways related to Cadherin-11 in LA-treated CD115(+) precursors. **b** RT-PCR analysis detected the mRNA levels of specific fibrotic markers in bone marrow at different timepoints after intratibially injection of MC-38 cells. **c**-**g** The primary CD115(+) precursors were isolated and transfected with Cadherin-11 siRNA or negative controls, then the cells were treated by LA for 72 h. The mRNA expression of each fibrotic marker was detected by RT-PCR. **h** After injecting MC-38 cells, LA was injected intraperitoneally for 5 days and Cadherin-11 siRNA was injected every 2 days. Then the primary CD115(+) precursors were isolated and fixed. Immunofluorescence analysis revealed the expression of Col5a2 in CD115(+) precursors sorted from mice in each group (left) and the quantification of percentage of Col5a2(+) cells (right) (Scale bar = 50 μm). *p < 0.05, **p < 0.01, ***p < 0.001

Interestingly, recent studies revealed that macrophages regulate fibrogenesis based on the expression of Cadherin-11 [29]. We thus wanted to determine whether the overexpression of Cadhein-11 in CD115(+) precursors induced by LA can also regulate collagen deposition. We first analyzed pathways involving Cdh11, and GO enrichment analysis showed that several extracellular matrix and collagen pathways were changed (Fig. 7a). RT-PCR analysis showed that the expression of several collagens, including Col5 and Col6, was increased at 14 days after tibial injection of MC-38; however, the expression of Collagen I was not significantly changed (Fig. 7b). Then, we sorted CD115(+) precursors, stimulated them with LA and transfected them with/without Cadherin-11 siRNA. The results showed that the mRNA levels of Col3a1, Col5a2 and Col6a3 were upregulated in the LA treatment group compared with the control group and that this increase was inhibited after transfection with Cadherin-11 siRNA (Fig. 7c-g). Immunofluorescence analysis of freshly isolated CD115(+) precursors proved that the expression of collagen 5 was upregulated after injection of LA and was downregulated by the combination of LA injection and Cadherin-11 siRNA transfection in vivo (Fig. 7h). Our data imply that CD115(+) precursors at least partially and selectively participate in fibrogenesis in tumors.

### PI3K pathway antagonists prevent osteolytic lesions in bone metastasis from CRC and lead to a better prognosis

**Fig. 8:**
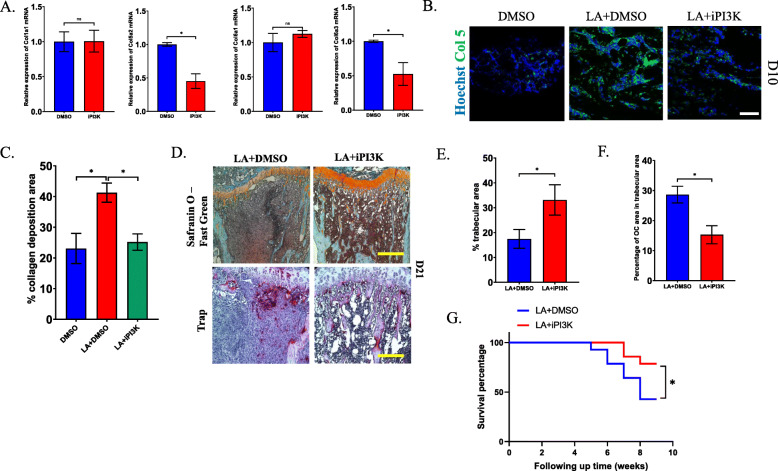
Inhibiting PI3K pathway attenuates the bone metastasis from CRC. **a** RT-PCR analysis detected the expression of fibrotic markers in CD115(+) precursors after treated by PI3K antagonist. **b** Representative images of expression of Collagen 5 in tibial cancellous bone in LA treated group with/without PI3K antagonist at 10 days post injection of MC-38 cells (Scale bar = 50 μm). **c** Quantification of area of Collagen 5 in (**b**). **d** Safranin O and fast green staining and trap staining analyzed the trabecular area and the TRAP(+) area in bone surface at 3 weeks after injection of MC-38 cells in LA as well as PI3K antagonist treated group vs. LA treated group (Scale bar = 50 μm). **e** The quantification of percentage of trabecula area and (**f**) the number of osteoclasts in (**d**). **g** Survival analysis in LA combined with PI3K antagonist treated group vs. LA-treated group in bone metastasis model of CRC. *p < 0.05, **p < 0.01, ***p < 0.001

Based on our findings, we next investigated whether inhibiting the PI3K pathway attenuates osteolytic lesions and tumor progression. The expression of Col5a2 and Col6a3 was downregulated in the PI3K inhibitor-treated group compared to the control group, but there was no effect on the mRNA levels of Col1a1 and Col6a1 (Fig. 8a). Moreover, immunofluorescence showed that the protein expression of Col5a2 in cancellous bone was increased in the LA-treated group compared to the control group and was attenuated by treatment with a PI3K inhibitor 10 days after injection of MC-38 (Fig. 8b and c). These data demonstrated that inhibiting PI3K can mostly reverse the fibrosis caused by LA. We also found that the progression of osteolytic lesions in bone metastasis was significantly prevented and that the number of osteoclasts was decreased in the PI3K inhibitor-treated group compared with the LA-treated group (Fig. 8d-f). Furthermore, the survival rate obviously increased after treatment with the PI3K inhibitor (Fig. 8g). Taken together, these data reveal that a PI3K inhibitor can prevent the progression of bone metastasis from CRC caused by LA.

## Discussion

The findings of this study reveal the indispensable role of LA in metastatic niche formation in bone metastasis from CRC and show that the PI3K pathway in osteoclast precursors plays a critical role in this pathological process. It is well known that the accumulation of LA in the tumor microenvironment creates an acidic environment, which largely affects cell behaviors and the progression of tumors. Interestingly, in addition to cancer cells, LA is closely associated with the modulation of the inflammatory microenvironment in tumors, especially monocytes and tumor-associated macrophages (TAMs) [30–34]. Generally, LA tends to facilitate the survival of cancer cells. The antitumor effects of some cell types, such as natural killer (NK) cells and monocytes, can be suppressed by the accumulation of LA [30–32, 35]. However, the role of LA in bone metastasis has rarely been studied. We found more severe bone absorption and a poorer prognosis after administration of LA in a model of bone metastasis from CRC. Consistent with this finding, the number of osteoclasts increased significantly after LA stimulation. The results demonstrated that LA participates in progressive bone destruction after CRC metastasis.

Surprisingly, unlike previous research, our data revealed that LA indirectly, but not directly, contributes to the accumulation of osteoclasts through proliferation, apoptosis or differentiation. CD4(+) T cells play an important role in this pathologic process. The level of CXCL10 in osteoclast precursors may increase after treatment with LA. Then, CXCL10 recruits CD4(+) T cells and promotes the production of RANKL through CXCR3 signaling. Finally, the elevation of RANKL promotes the osteoclastogenesis of CD115(+) precursors. The whole process forms a positive-feedback loop, which causes the progression of bone resorption in bone from of CRC. Interestingly, the ability of CD4(+) T cells to produce RANKL was recognized previously, and RANKL derived from CD4(+) T cells induces the differentiation of osteoclast precursors in rheumatic arthritis and causes bone resorption in joints. Based on our findings, CXCL10-mediated osteoclastogenesis through CD4(+) T cells may occur in a variety of destructive bone diseases and could be a potential therapeutic target.

Previous studies have shown that LA can promote the recruitment and invasion of cervical cancer cells [36]; inconsistently, we found that the recruitment of osteoclast precursors treated with LA was inhibited in this study, indicating that LA has heterogeneous effects on different cell types. Transcriptomic analysis identified Cdh11, which encodes an adhesive protein called Cadherin-11, as a key regulator of adhesion and recruitment of osteoclast precursors mediated by LA. Cadherin-11 has been found to have multiple functions, including cell-cell adhesion, recruitment, angiogenesis and collagen deposition [28, 37–40]. However, the role of cadherin-11 in adhesion and recruitment remains controversial [41, 42]. Our study revealed that LA can upregulate the expression of Cadherin-11 and mediate adhesion between osteoclast precursors while preventing recruitment. This result is consistent with our finding that LA prevents the recruitment of osteoclast precursors. Previous studies revealed that two cells expressing Cadherin-11 can adhere to each other [29]. Based on our findings, we speculate that two osteoclast precursors expressing Cadherin-11 may anchor each other to enhance adhesion and inhibit mobility, which facilitates the fusion of osteoclast precursors to form osteoclasts; however, the details of this process need to be further investigated.

In addition to adhesion, Cadherin-11 has been reported to be associated with collagen production [41, 43–45]. Our findings support this hypothesis. Cadherin-11 mediated LA-induced collagen expression in CD115(+) precursors, which may participate in collagen deposition in the tumor microenvironment. Interestingly, the expression of fibrosis markers was selective, and the expression of two main fibrosis markers, Col1a1 and Col3a1, was not upregulated after treatment with LA and was not impacted by the inhibition of Cadherin-11. Although the mechanism is still unclear, considering that Collagen I is one of the most important collagens in the formation of trabeculae, our results imply that LA may not only stimulate bone resorption but also lead to the reconstruction of collagens in cancellous bone for adaptation to tumor progression and prevention of bone formation.

Interestingly, we revealed the critical role of the PI3K-AKT pathway in the LA-mediated regulation of CXCL10 and Cadherin-11. Several studies have revealed that the PI3K pathway participates in the regulation of CXCL10 [21, 46] and that inhibiting the PI3K pathway downregulates the production of CXCL10 and affects CXCL10 gene promoter activity, which is consistent with the findings of this study. Furthermore, a previous study showed that CREB is involved in the expression of CXCL10 [23]. Here, we revealed that inhibiting the transcription factor CREB directly can downregulate the expression of CXCL10. Previous studies have also demonstrated that the PI3K-AKT pathway can be activated in monocyte/macrophage linear cells in colorectal cancer through extracellular signaling [47–49], which promotes the progression of tumors. In our study, we found that lactate is a new regulator that activates the PI3K-AKT pathway in osteoclast precursors. Inhibiting the PI3K-AKT pathway using LY294002 can attenuate LA-mediated cell adhesion, fibrosis and bone resorption and thus prolong survival, suggesting that it is a reasonable target for the treatment of bone metastasis from CRC.

## Conclusions

In conclusion, our findings reveal that LA can promote the expression of CXCL10 and Cadherin-11 in CD115(+) precursors through the PI3K-AKT pathway, which thus facilitates the formation of osteolytic lesions and the metastatic niche. In addition, CXCL10 and Cadherin-11 are regulated by the activation of CREB and mTOR, respectively. Overexpression of CXCL10 can promote the differentiation of osteoclast precursors indirectly through the recruitment of CD4(+) T cells and cause bone resorption. On the other hand, Cadherin-11 promotes adhesion between osteoclast precursors and increases the production of specific collagens, which facilitates fibrotic changes in the tumor microenvironment. We further showed that blockade of the PI3K-AKT pathway efficiently prevents the progression of metastasis caused by lactate and could be a potential therapeutic target for bone metastasis from CRC.

## Supplementary Information

**Additional file 1: Figure S1**. LA does not contribute to the proliferation, apoptosis and differentiation of CD115(+) osteoclast precursors directly. (A) Strategy for sorting CD115(+) precursors from bone marrow. (B) Representative images for Ki67 positive cells in LA treated group and control group (left) and quantification of percentage of Ki67 positive osteoclast precursors (right). (C) Statistics analysis of BrdU (+) cells in CD115 (+) precursors after stimulated by LA and M-CSF in vitro for 3 days and (D) without stimulation by M-CSF. (E) Annexin-V/PI analysis tested the percentage of apoptosis of CD115 (+) precursors after treatment with LA in vitro for 3 days. (F) TRAP relative activity assay detected the Trap activity in CD115(+) cells after stimulated by LA and RANKL for 4 days. *p < 0.05, **p < 0.01, ***p < 0.001. **Figure S2**. CXCL10 does not directly contribute to the differentiation of osteoclast precursors. (A) TRAP staining showed the TRAP (+) osteoclasts (left) and quantification of the number of TRAP (+) osteoclasts (right) (Scale bar = 50 μm). (B) TRAP relative activity analysis showed the relative TRAP activity in osteoclastogenesis of CD115 (+) precursors between LA-treated group and control group. (C) The mRNA expression of CXCR3 in CD115 (+) precursors in LA-treated group comparing with control group. *p < 0.05, **p < 0.01, ***p < 0.001.

**Additional file 2: Table S1.** Transcriptome profiling showing differentially expressed chemokines in CD115(+) precursors stimulated by LA.

## Data Availability

All data generated or analyzed during this study are included in this published article and its supplementary information files.

## Abbreviations

CRC: Colorectal cancer
OC: Osteoclast
qRT-PCR: Real time quantitative reverse transcription PCR
LA: Lactate
3-OBA: 3-hydroxy-butyrate
FACS: Fluorescence activated cell sorter
GM: Growth medium
DMEM: Dulbecco’s modified eagle medium
α-MEM: α-minimal essential medium
FBS: Fetal bovine serum
CSF: Colony stimulating factor
PFA: Paraformaldehyde
ANOVA: One-way analysis of variance
PI3K: Phosphoinositide 3-kinase
BrdU: Bromodeoxyuridine
Trap: Tartrate resistant acid phosphatase
RANKL: Receptor Activator of Nuclear Factor-κ B Ligand
FLS: Fibroblast-like synovial
MSC: Mesenchymal stem cell
Cxcl10: Chemokine (C-X-C motif) ligand 10
CXCR3: C-X-C Motif Chemokine Receptor 3
