# Supplementary figures and images for: Lactic acid promotes metastatic niche formation in bone metastasis of colorectal cancer
Source: Cell Commun Signal. 2021 Jan 21;19:9. doi: 10.1186/s12964-020-00667-x (PMC7818572; doi:10.1186/s12964-020-00667-x)

## Slide 1
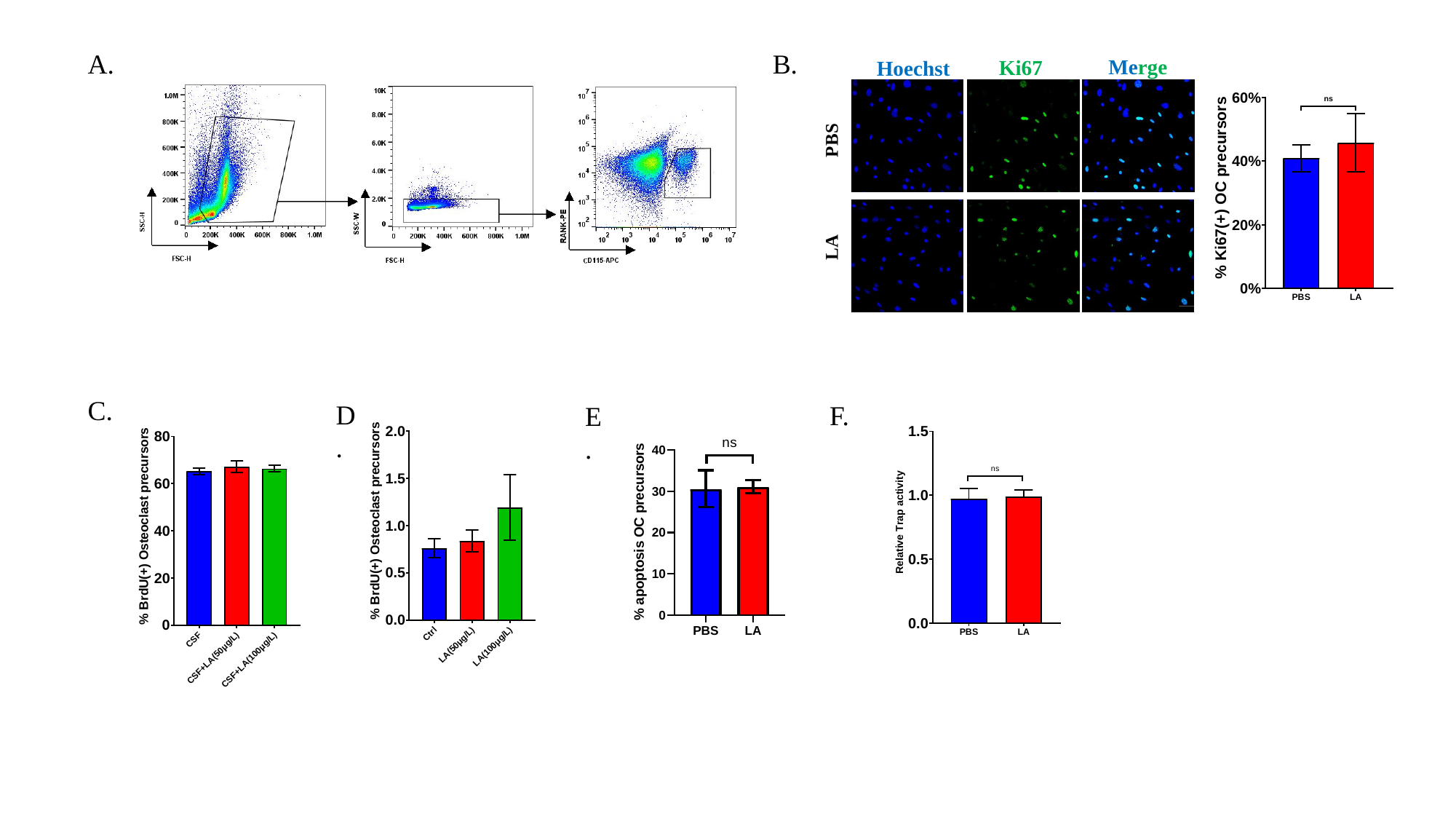

A.
B.
Merge
Ki67
Hoechst
PBS
LA
C.
D.
F.
E.

## Slide 2
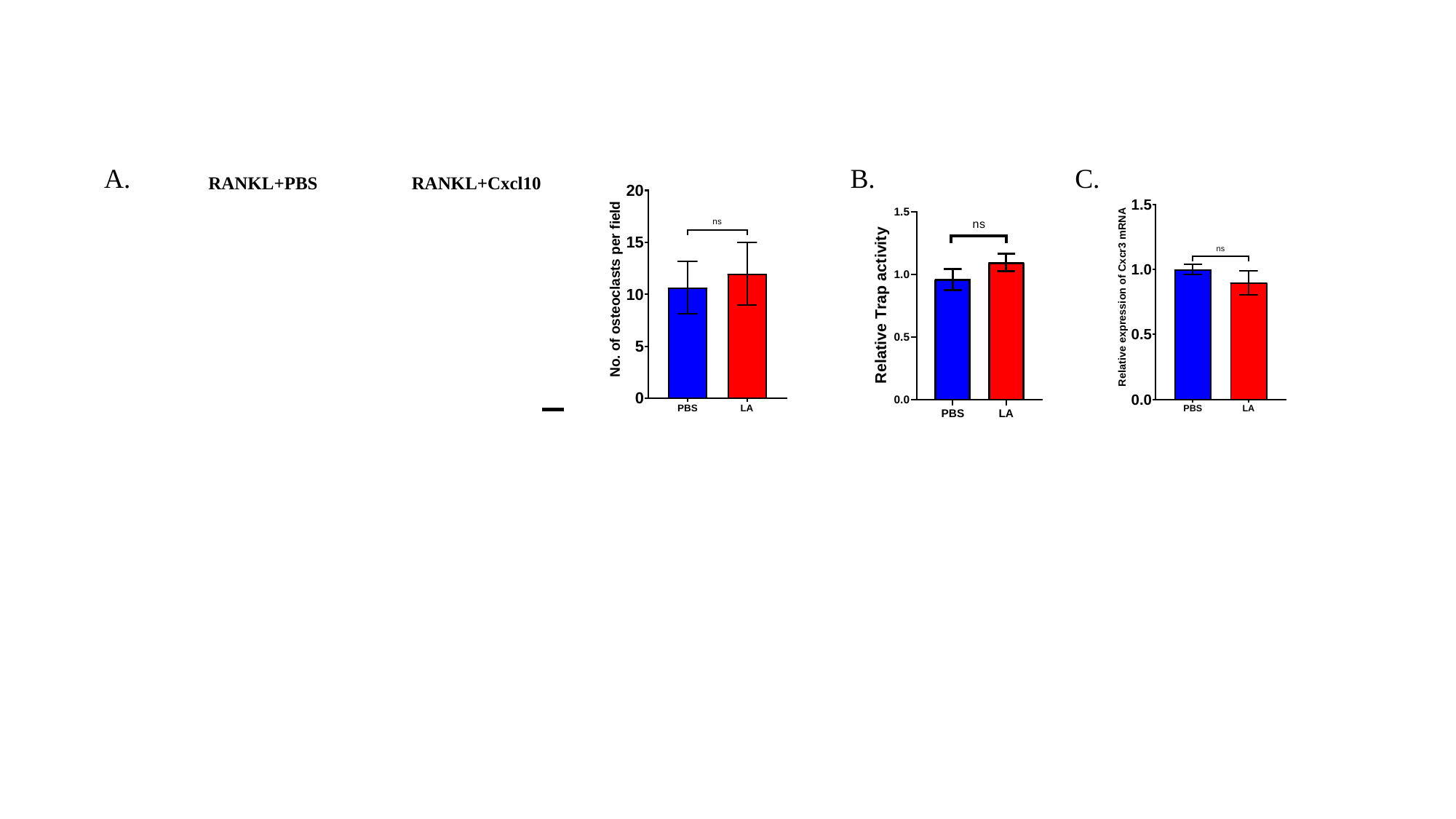

B.
C.
A.
RANKL+Cxcl10
RANKL+PBS

Supplement: Supplementary file 2 — Additional file 1: Figure S1. LA does not contribute to the proliferation, apoptosis and differentiation of CD115(+) osteoclast precursors directly. (A) Strategy for sorting CD115(+) precursors from bone marrow. (B) Representative images for Ki67 positive cells in LA treated group and control group (left) and quantification of percentage of Ki67 positive osteoclast precursors (right). (C) Statistics analysis of BrdU (+) cells in CD115 (+) precursors after stimulated by LA and M-CSF in vitro for 3 days and (D) without stimulation by M-CSF. (E) Annexin-V/PI analysis tested the percentage of apoptosis of CD115 (+) precursors after treatment with LA in vitro for 3 days. (F) TRAP relative activity assay detected the Trap activity in CD115(+) cells after stimulated by LA and RANKL for 4 days. *p < 0.05, **p < 0.01, ***p < 0.001. Figure S2. CXCL10 does not directly contribute to the differentiation of osteoclast precursors. (A) TRAP staining showed the TRAP (+) osteoclasts (left) and quantification of the number of TRAP (+) osteoclasts (right) (Scale bar = 50 μm). (B) TRAP relative activity analysis showed the relative TRAP activity in osteoclastogenesis of CD115 (+) precursors between LA-treated group and control group. (C) The mRNA expression of CXCR3 in CD115 (+) precursors in LA-treated group comparing with control group. *p < 0.05, **p < 0.01, ***p < 0.001. [file 12964_2020_667_MOESM2_ESM.pptx]
