# Supplementary material for: Lactic acid promotes metastatic niche formation in bone metastasis of colorectal cancer
Source: Cell Commun Signal. 2021 Jan 21;19:9. doi: 10.1186/s12964-020-00667-x (PMC7818572; doi:10.1186/s12964-020-00667-x)
Supplement: Supplementary file 3 — Additional file 2: Table S1. Transcriptome profiling showing differentially expressed chemokines in CD115(+) precursors stimulated by LA. [file 12964_2020_667_MOESM3_ESM.docx]

Table. S1 Transcriptome profiling showing differentially expressed chemokines in CD115(+) precursors stimulated by LA

| **Gene_id** | **Gene name** | **FC(LA/Ctrl)** | **Padjust** |
| --- | --- | --- | --- |
| ENSMUSG00000034855 | Cxcl10 | 2.172 | 0 |
| ENSMUSG00000029417 | Cxcl9 | 2.028 | 1.06E-34 |
| ENSMUSG00000018593 | Sparc | 0.061 | 7.41E-12 |
| ENSMUSG00000044701 | Il27 | 2.124 | 2.94E-07 |
| ENSMUSG00000023885 | Thbs2 | 0.071 | 8.72E-07 |
| ENSMUSG00000029371 | Cxcl5 | 0.309 | 0.04245 |
| ENSMUSG00000031480 | Thsd1 | 0.483 | 0.044204 |
